# Supplementary material for: In vivo detection of antisense HIV-1 transcripts in untreated and ART-treated individuals
Source: Life Sci Alliance. 2025 Jul 14;8(9):e202503204. doi: 10.26508/lsa.202503204 (PMC12260654; doi:10.26508/lsa.202503204)
Supplement: Supplementary file 1 [file LSA-2025-03204_TableS1.docx]

**Table S1.** **Primers for AST cDNA synthesis and PCR amplification.**

| **Primer Name** | **Primer Sequence**  **(5ʹ→3ʹ)** | **Primer Use** |
| --- | --- | --- |
| Tag Fwd**^A^** | **CTGATCTAGAGGTACCGGATCC** | Tag acting as Fwd primer |
| AST cDNA | **CTGATCTAGAGGTACCGGATCC**AACATGTGGCAGGAAGTAGG | *env* AST cDNA primer |
| Env cDNA | **CTGATCTAGAGGTACCGGATCC**ACATCTAATTTGTCCACTGA | *env* cDNA primer |
| 1849Tag+ | **CTGATCTAGAGGTACCGGATCC**GATGACAGCATGTCAGGGAG | *gag/pol* AST cDNA primer |
| 3996Tag+ | **CTGATCTAGAGGTACCGGATCC**CATCTAGCTTTGCAGGATTCG | *pol/vif* AST cDNA primer |
| G00-Tag+ | **CTGATCTAGAGGTACCGGATCC**GACTAGCGGAGGCTAGAAG | *gag* AST cDNA primer |
| AST-OF | **CTGATCTAGAGGTACCGGATCC** | *env* AST PCR1**^B^** |
| AST-OR | TGGTACTAGCTTGTAGCACCATCC |  |
| AST-IF | AGCAGAACAATTTGCTGAGGGC | *env* AST PCR 2**^C^** |
| AST-IR | GTCATTGGTCTTAAAGGTACCTGAGG |  |
| 1849+ | GGATCCGATGACAGCATGTCAGGGAG | *gag/pol* AST PCR1**^D^** |
| 3500- | CTATTAAGTCTTTTGATGGGTCATAA |  |
| 1870+ | GAGTTTTGGCTGAAGCAATGAG | *gag/pol* AST PCR2**^E^** |
| 3410- | CTGTTAGTGGTATTACTTCTGTTAGTGCTT |  |
| 5270- | CTGACCCAAATGCCAGTCTC | *pol/vif* AST PCR1**^F^** |
| 4133+ | GGAAAAGGTCTATCTGGCATG | *pol/vif* AST PCR2**^G^** |
| 5248- | TCTCCTGTATGCAGACCCCA |  |
| G01 | AGGGGTCGTTGCCAAAGA | *gag* AST PCR1**^H^** |
| G10 | CAGTATTAAGCGGGGGAGAATT | *gag* AST PCR2**^I^** |
| G15 | CTTTGCCACAATTGAAACACTT |  |
| envB5out | TAGAGCCCTGGAAGCATCCAGGAAGT | *env* PCR1**^J^** |
| 9538r | AGAGAGACCCAGTACAGGCAAAA |  |
| envB5in | TTAGGCATCTCCTATGGCAGGAAGAAG | *env* PCR2**^K^** |
| 9418r | CAAGCTCGATGTCAGCAGTTCT |  |

**^A^** Exogenous oligo-tag in black bold

**^B^** *env* AST PCR1: 94°C for 2 min, 45 cycles of 94°C for 15 sec, 60°C for 15 sec, and 68°C for 30 sec

**^C^** *env* AST PCR2: 94°C for 2 min, 40 cycles of 94°C for 15 sec, 60°C for 15 sec, and 68°C for 20 sec

**^D^** *gag*/*pol* AST PCR1: 94°C for 2 min, 45 cycles of 94°C for 15 sec, 60°C for 15 sec, and 68°C for 30 sec

**^E^** *gag*/*pol* AST PCR2: 94°C for 2 min, 40 cycles of 94°C for 15 sec, 60°C for 15 sec, and 68°C for 30 sec

**^F^** *pol*/*vif* AST PCR1: 94°C for 2 min, 45 cycles of 94°C for 15 sec, 60°C for 15 sec, and 68°C for 20 sec

**^G^** *pol*/*vif* AST PCR2: 94°C for 2 min, 40 cycles of 94°C for 15 sec, 60°C for 15 sec, and 68°C for 20 sec

**^H^** *gag* AST PCR1: 94°C for 2 min, 45 cycles of 94°C for 15 sec, 60°C for 15 sec, and 68°C for 25 sec

**^I^** *gag* AST PCR2: 94°C for 2 min, 40 cycles of 94°C for 15 sec, 60°C for 15 sec, and 68°C for 20 sec

**^J^** *env* PCR1: 94°C for 2 min, 45 cycles of 94°C for 15 sec, 60°C for 30 sec, and 68°C for 3 min

**^K^** *env* PCR2: 94°C for 2 min, 40 cycles of 94°C for 15 sec, 60°C for 30 sec, and 68°C for 20 sec
