# Supplementary material for: In vivo detection of antisense HIV-1 transcripts in untreated and ART-treated individuals
Source: Life Sci Alliance. 2025 Jul 14;8(9):e202503204. doi: 10.26508/lsa.202503204 (PMC12260654; doi:10.26508/lsa.202503204)
Supplement: Supplementary file 2 [file LSA-2025-03204_TableS2.docx]

**Table S2.** **Primers used for AST controls.**

| **Primer Name** | **Primer Sequence** **(5ʹ→3ʹ)** |
| --- | --- |
| AST-NEF2R | GTCATTGGTCTTAAAGGTACCTGAGG |
| AST-1586R | AGCAGAACAATTTGCTGAGGGC |
| AST-SEQ2R | GGTGAATATCCCTGCCTAACTCTAT |
| AST-SEQ2F | GGTTTAACATAACAAATTGGCTGTGGTATATAA |
| AST-SEQ3F | ATGGGTGGCAAGTGGTCAAA |
| Cloning Analysis Fwd | ACCTGCCAACCAAAGCGAGAAC |
| Cloning Analysis Rev | TCAGGGTTATTGTCTCATGAGCG |
| Rev16 | ATGGGAGGGGCATACATTGCT |
| Rev17 | CCTGGAGCTGTTTAATGCCCCAGAC |
| M13 Fwd | GTAAAACGACGGCCAGT |
| M13 Rev | CAGGAAACAGCTATGAC |
