# Supplementary material for: In vivo detection of antisense HIV-1 transcripts in untreated and ART-treated individuals
Source: Life Sci Alliance. 2025 Jul 14;8(9):e202503204. doi: 10.26508/lsa.202503204 (PMC12260654; doi:10.26508/lsa.202503204)
Supplement: Supplementary file 3 [file LSA-2025-03204_TableS3.docx]

**Table S3. Digital PCR Primers and Probes.**

| **Primer Name** | **Primer/Probe Sequence** **(5ʹ→3ʹ)** | **Primer Use** |
| --- | --- | --- |
| AST-Tag | **ATCTAGAGGTACCGGATCCAAC** | *env* AST |
| AST Rev-qPCR | TGATGAACATCTAATTTGTCCACTGA |  |
| AST Probe | /56-FAM/AGCAATGT/ZEN/ATGCCCCTCCCA/3IaBkFQ/ |  |
| AST-Tag | **ATCTAGAGGTACCGGATCCACA** | *env* |
| *env* Rev-qPCR | TTTAATTGTGGAGGGGAATTTTTCTA |  |
| env Probe | /56-FAM/TGGGAGGGGCATA/ZEN/CATTGCT/3IaBkFQ/ |  |
| LTR U5 Fwd | CTTAAGCCTCAATAAAGCTTGCC | LTR U5 |
| LTR U5 Rev | GGATCTCTAGTTACCAGAGTC |  |
| LTR U5 Probe | /5’HEX/AGTAGTGTG/ZEN/TGCCCGTCTG/3IaBkFQ/ |  |
| RRE Fwd | GCAGAGAGAAAAAAGAGC | RRE |
| RRE Rev | GCCTGTACCGTCAGC |  |
| RRE Probe | /5’HEX/TTCCTTGGG/ZEN/TTCTTGGGAGCAG/3IaBkFQ/ |  |
| iCAD Fwd | TTTGGAAAGGACCAGCAAA | IN DNA  Assay  (iCAD) |
| iCAD Rev | CCTGCCATCTGTTTTCCA |  |
| iCAD Probe | /56-FAM/AAAGGTGAA/ZEN/GGGGCAGTAGTAATACA/3IaBkFQ/ |  |

Exogenous oligo-tag sequence in bold
