# Supplementary material for: In vivo detection of antisense HIV-1 transcripts in untreated and ART-treated individuals
Source: Life Sci Alliance. 2025 Jul 14;8(9):e202503204. doi: 10.26508/lsa.202503204 (PMC12260654; doi:10.26508/lsa.202503204)
Supplement: Supplementary file 4 [file LSA-2025-03204_TableS4.docx]

**Table S4. Sequencing Primers.**

| **Primer Name** | **Primer Sequence** **(5ʹ→3ʹ)** | **Primer Use** |
| --- | --- | --- |
| AST-Seq2 Rev | GGTGAATATCCCTGCCTAACTCTAT | *env* AST |
| AST-Seq2 Fwd | GGTTTAACATAACAAATTGGCTGTGGTATATAA |  |
| AST-Seq3 Fwd | ATGGGTGGCAAGTGGTCAAA |  |
| AST-IF | AGCAGAACAATTTGCTGAGGGC |  |
| AST-IR | GTCATTGGTCTTAAAGGTACCTGAGG |  |
| 2030+ | TGTTGGAAATGTGGAAAGGAAGGAC | *gag/pol* AST |
| 2600+ | ATGGCCCAAAAGTTAAACAATGGC |  |
| 2610- | TTCTTCTGTCAATGGCCATTGTTTAAC |  |
| 3330- | TTGCCCAATTCAATTTTCCCACTAA |  |
| Poli9D | AAAATTAGCAGGAMGATGGCCAG | *pol/vif* AST |
| Poli10B | TATTCATAGATTCYACTACTCCTTG |  |
| 4133+ | GGAAAAGGTCTATCTGGCATG |  |
| 5248- | TCTCCTGTATGCAGACCCCA |  |
| G30 | CAGTAGCAACCCTCTATTGTGT | *gag* AST |
| G25 | ATTGCTTCAGCCAAAACTCTTGC |  |
| G10 | CAGTATTAAGCGGGGGAGAATT |  |
| G15 | CTTTGCCACAATTGAAACACTT |  |
| For16 | TTTAATTGTGGAGGAGAATTTTTCTA | *env* |
| For17 | AGCAGCAGGAAGCAGTATGGGCGC |  |
| For18 | CATATCAAATTGGCTGTGGTATAT |  |
| Rev15 | CTGCCATTTAACAGCAGTTGAGTTGA |  |
| Rev16 | ATGGGAGGGGCATACATTGCT |  |
| envB5in | TTAGGCATCTCCTATGGCAGGAAGAAG |  |
| envB5out | TAGAGCCCTGGAAGCATCCAGGAAGT |  |
| 9418r | CAAGCTCGATGTCAGCAGTTCT |  |
