# Supplementary material for: In vivo detection of antisense HIV-1 transcripts in untreated and ART-treated individuals
Source: Life Sci Alliance. 2025 Jul 14;8(9):e202503204. doi: 10.26508/lsa.202503204 (PMC12260654; doi:10.26508/lsa.202503204)
Supplement: Supplementary file 5 [file LSA-2025-03204_TableS5.docx]

**Table S5. Donor specific primers.**

| **Primer Name** | **Primer Sequence**  **(5ʹ→3ʹ)** |
| --- | --- |
| 2669_ASTI-F | AGCAGAACAATTTGCTGAG**R**GC |
| 2669_ASTI-R | GTCATTGGTCTTAAAGGTACCTG**R**GG |
| 2669 AST-R-qPCR | TGATGAACATCTAATT**A**GTCC**TT**TGA |
| 2669_*env*-Tagged | **CTGATCTAGAGGTACCGGATCC**TGATGAACATCTAATT**A**GTCC**TT**TGA |
| 1079_ASTR-qPCR | TGATGAACATCTAATTT**S**TCC**M**CTGA |
| 1079_ *env* -Tagged | **CTGATCTAGAGGTACCGGATCC**ACATCTAATTT**S**TCC**M**CTGA |
| 1683_AST-Tagged | **CTGATCTAGAGGTACCGGATCC**AACATGTGGCA**AG**AA**A**TAGG |
| 1683_AST-OR | TGGTACTAGCTTGTAGCACCA**C**CC |
| 1683_AST-IF | AGCAGAACAATTTGCTGAG**A**GC |
| 1683_AST-R-qPCR | TGATGAACATCTAAT**CA**GTCC**G**CTGA |
| 1683_ *env* -Tagged | **CTGATCTAGAGGTACCGGATCC**ACATCTAAT**CA**GTCC**G**CTGA |
| 1683_ *env* F-qPCR | ACAAATTATAAACATGTGGCA**AG**AA**A**TAGG |
| 291_AST-R | TGATGAACA**RY**TAATTT**K**TCC**TY**TGA |
| 1508_AST-Tagged | **CTGATCTAGAGGTACCGGATCC**AA**T**ATGTGGCAG**GG**AGTAGG |
| 1508_AST-R | TGATGAACA**K**CTAATTTGTCC**KYK**GA |
| 1508_AST-P | /56-FAM/AGC**C**ATGTA/ZEN/TGCCCCTCCCA/3IaBkFQ/ |
| 3611_AST-Tagged | **CTGATCTAGAGGTACCGGATCC**AACATGTGGCA**AG**AAGTAGG |
| 3611_AST-R | TGATG**C**ACA**G**CTAATT**Y**GTCC**T**CTGA |
| 3611_AST-P | /56-FAM/A**G**GCAATGT/ZEN/ATGCCCCTCCCA/3IaBkFQ/ |
| 1226_AST-Tagged | **CTGATCTAGAGGTACCGGATCC**AACATGTGGCA**AC**AAGTAGG |
| 1226_AST-R | TGATGAACA**G**CTAAT**C**TGTCC**T**C**G**GA |
| 1775_AST-Tagged | **CTGATCTAGAGGTACCGGATCC**AACATGTGGCA**R**AAAGTAGG |
| 1775_AST-R | TGATGAACAT**T**TAATTTGTCC**T**CTGA |
| 477_AST-R | TGATGAACA**Y**CTAATTAGTCCACTGA |

Exogenous oligo-tag sequence in black bold

Bold red nucleotide reflects donor specific base

Degenerate bases: R (A/G), S (S/G), M (A/C), Y (C/T), and K (G/T) following IUPAC nucleotide code
