# Supplementary material for: In vivo detection of antisense HIV-1 transcripts in untreated and ART-treated individuals
Source: Life Sci Alliance. 2025 Jul 14;8(9):e202503204. doi: 10.26508/lsa.202503204 (PMC12260654; doi:10.26508/lsa.202503204)
Supplement: Supplementary file 7 [file LSA-2025-03204_TableS6.docx]

**Table S6.** **Levels of HIV-1 AST in ART-treated and untreated donors.**

| **Participant Identifier (PID)** | **Duration on ART at sampling** | **Estimated number of infected PBMC assayed^B^** | **Number of HIV-1 AST molecules detected** | **Number of HIV-1 AST molecules per 100 infected PBMC** |
| --- | --- | --- | --- | --- |
| 1079 | 12.8 years | 80 | 9 | 11 |
| 1683 | 5.4 years | 188 | 32 | 17 |
| 2669 | 4.3 years | 90 | 19 | 21 |
|  | 5.5 years | 65 | 20 | 31 |
|  | 2 weeks**^A^** | 88 | 34 | 39 |
|  | 1 month**^A^** | 45 | 31 | 69 |
| **Median** | | **84** | **26** | **26** |
| **IQR** | | **60-115** | **17-33** | **16-47** |
| 291 | 0 | 104 | 2 | 2 |
| 477 | 0 | 83 | 2 | 2 |
| 1508 | 0 | 22 | 1 | 5 |
| 1775 | 0 | 40 | 13 | 33 |
| 3611 | 0 | 244 | 1 | ≤0.4**^C^** |
| **Median** | | **83** | **2** | **2** |
| **IQR** | | **31-174** | **1-8** | **1-19** |

**^A^** After 5.5 years on ART, the participant had an unexpected ART interruption for approximately 4 weeks They reinitiated ART with the first timepoint post-ART interruption at 2 weeks with low but detectable HIV-1 plasma viremia. Then 1 month post-ART interruption with plasma viremia suppressed

**^B^** Estimated the number of HIV DNA levels using the integrase cell-associated single-copy DNA (iCAD) assay (Hong et al. 2016) adapted for *env* using RRE (Bruner et al. 2019)

**^C^** Detectable but below the cut-off
