# Supplementary material for: In vivo detection of antisense HIV-1 transcripts in untreated and ART-treated individuals
Source: Life Sci Alliance. 2025 Jul 14;8(9):e202503204. doi: 10.26508/lsa.202503204 (PMC12260654; doi:10.26508/lsa.202503204)
Supplement: Supplementary file 8 [file LSA-2025-03204_TableS7.docx]

**Table S7.** **Levels of HIV-1 *env* transcripts in ART-treated and untreated donors.**

| **Participant Identifier (PID)** | **Duration on ART at sampling** | **Estimated number of infected PBMC assayed^B^** | **Number of HIV-1 *env* transcripts detected** | **Number of HIV-1 *env* transcripts per 100 infected PBMC** |
| --- | --- | --- | --- | --- |
| 1079 | 12.8 years | 80 | 5 | 6 |
| 1683 | 5.4 years | 94 | 3 | 3 |
| 2669 | 4.3 years | 90 | 17 | 19 |
|  | 5.5 years | 130 | 16 | 12 |
|  | 2 weeks**^A^** | 125 | 30 | 24 |
|  | 1 month**^A^** | 90 | 24 | 27 |
| **Median** | | **92** | **17** | **16** |
| **IQR** | | **88-126** | **5-26** | **5-25** |
| 291 | 0 | 40 | 1 | 3 |
| 477 | 0 | 120 | 16 | 13 |
| 1508 | 0 | 86 | ≤0.01^C^ | ≤1^C^ |
| 1775 | 0 | 100 | 142 | 142 |
| 3611 | 0 | 56 | 4 | 7 |
| **Median** | | **86** | **4** | **7** |
| **IQR** | | **48-110** | **1-79** | **2-78** |

**^A^** After 5.5 years on ART, the participant had an unexpected ART interruption for approximately 4 weeks They reinitiated ART with the first timepoint post-ART interruption at 2 weeks with low but detectable HIV-1 plasma viremia. Then 1 month post-ART interruption with plasma viremia suppressed

**^B^** Estimated the number of HIV DNA levels using the integrase cell-associated single-copy DNA (iCAD) assay (Hong et al. 2016) adapted for *env* using RRE (Bruner et al. 2019)

**^C^** HIV-1 *env* transcripts were not detected. Potentially 1/number of infected cells assayed was used as an upper limit, and then normalized to 100 infected PBMC
